# White Matter Correlates of Psychopathic Traits in a Japanese Community Sample: Sex Matters!

**DOI:** 10.1111/ejn.70177

**Published:** 2025-07-14

**Authors:** S. C. Chester, J. C. Rogers, T. Ogawa, M. Terao, R. Nakai, N. Abe, S. A. De Brito

**Affiliations:** ^1^ Centre for Human Brain Health University of Birmingham Birmingham UK; ^2^ Institute for Mental Health, School of Psychology University of Birmingham Birmingham UK; ^3^ Birmingham Centre for Neurogenetics University of Birmingham Birmingham UK; ^4^ Institute for the Future of Human Society Kyoto University Kyoto Japan; ^5^ Division of Transdisciplinary Sciences, Graduate School of Advanced Science and Technology Japan Advanced Institute of Science and Technology Nomi Japan; ^6^ Centre for Developmental Science, School of Psychology University of Birmingham Birmingham UK

**Keywords:** diffusion tensor imaging, psychopathy, sex differences, tract‐based spatial statistics, white matter

## Abstract

Psychopathy is a personality disorder marked by distinct interpersonal, affective and behavioural abnormalities. Relatively little is known about associations between psychopathic traits and white matter (WM) microstructure among community samples, as most diffusion tensor imaging (DTI) research has focused on small clinical and forensic samples. These studies are also often limited to males from Western populations, leaving gaps in the understanding of females and non‐Western populations. This study uses DTI data from a large community sample (*n* = 97) of well‐functioning Japanese adults (45 males, aged 21–39 years). We used tract‐based spatial statistics to investigate WM microstructural integrity (whole brain corrected using threshold‐free cluster enhancement (TFCE), *p* < 0.05). Psychopathy traits were measured using the Self‐Report Psychopathy Scale (SRP‐SF). Region of interest analysis showed that callous affect scores negatively correlated with fractional anisotropy and axial diffusivity in the left cingulum. Furthermore, analysis across the entire white matter skeleton revealed that sex acted as a moderator in the association between psychopathy trait scores and mean diffusivity (MD) in the right superior longitudinal fasciculus, anterior thalamic radiation, and corpus callosum. The interaction showed that psychopathy trait scores were positively associated with MD in females, whereas the opposite pattern was observed in males. Our findings offer novel insights into the white matter correlates of psychopathic traits.

## Graphical abstract

This study examines associations between psychopathic traits and WM microstructure.

Callous affect scores negatively correlate with WM integrity in the left cingulum.

Sex moderates the association between psychopathic traits and WM integrity.

Sex‐specific results offer new perspectives on psychopathy in non‐Western community samples.

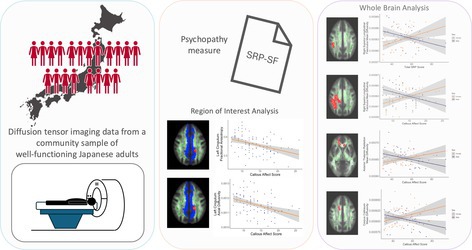

**Abbreviations**
ad
: axial diffusivity
ASB: antisocial behaviour
ASPD: antisocial personality disorder
CA: callous affect
CD: conduct disorder
DTI: diffusion tensor imaging
EL: erratic lifestyle
FA: fractional anisotropy
FWE: familywise error
GMV: grey matter volume
IM: interpersonal manipulation
MD: mean diffusivity
PCL‐R: psychopathy checklist revised
RD: radial diffusivity
ROI: region of interest
SD: standard deviation
SRP‐4: Self‐Report Psychopathy Scale Fourth Edition
SRP‐SF: Self‐Report Psychopathy Scale Short Form
TBSS: tract‐based spatial statistics
TFCE: threshold‐free cluster enhancement
WEIRD: Western, Educated, Industrialised, Rich and Democratic
WM: white matter

## 1 Introduction

Psychopathy is a personality disorder that manifests as a syndrome characterised by prominent affective and interpersonal deficits and persistent patterns of disruptive and antisocial behaviours (ASBs) (Hare and Neumann 2009). Although extreme presentations of psychopathy may be relatively rare (~1% of the general population), individuals with the disorder account for a disproportionate number of criminal offences and societal costs and place an enormous emotional burden on their victims (Anderson and Kiehl 2014; De Brito et al. 2021). This personality disorder is highly predictive of adverse clinical and behavioural outcomes such as criminal and violent offending, recidivism and poor treatment outcomes (Hare and Neumann 2008; Hare and Neumann 2009; Salekin et al. 1997). Factor analytic work has shown that psychopathy traits, as operationalised by the psychopathy checklist revised (PCL‐R; Hare 1991) (considered the ‘gold standard’ in the assessment of psychopathy), broadly coalesce around two dimensions (Hare and Neumann 2008; Hare and Neumann 2009); Factor 1 items index interpersonal and affective traits such as egocentricity, deception, low empathy, callousness and manipulation of others whereas Factor 2 relates to impulsive and antisocial traits such as social deviance, criminality and impulsivity (Hare and Neumann 2008; Hare and Neumann 2009). These factors can be further subdivided into four distinct personality and behaviour facets: interpersonal (Facet 1); affective (Facet 2); erratic lifestyle (EL) (Facet 3) and antisocial (Facet 4) (Vitacco et al. 2005).

In recent years, there has been a growing body of work focusing on the associations between psychopathy, its subcomponent facets and white matter microstructure in order to investigate disrupted brain circuitry, which may underlie the disorder (Johanson et al. 2020; Pujol et al. 2019; Waller et al. 2017). Much of the literature in this field has used diffusion tensor imaging (DTI) to investigate fractional anisotropy (FA), a scalar value providing an index for the degree of diffusion of water molecules within tracts in the brain (ranging from 0, isotropic, to 1, anisotropic). Calculating the degree of anisotropy provides a measure of tissue microstructure that occurs naturally as diffusion is restricted by cellular structures (such as cell membranes and fibres) (Le Bihan 2003; Mueller et al. 2015). Other commonly used DTI indices of white matter microstructure include axial diffusivity (ad), which reflects the diffusivity along the longest/principal axis and is assumed to point in the direction of the fibre tract; radial diffusivity (RD), which represents the average diffusion along the two shortest medium and minor axes; and mean diffusivity (MD), which provides the average diffusion of all three tensor dimensions (Figley et al. 2022). These metrics can then be used to reveal subtle differences in microstructural features, fibre bundle directions and differences in fibre density, axonal diameter and degree of myelination in different pathological conditions (Le Bihan 2003; Mueller et al. 2015; Winklewski et al. 2018).

Research in psychopathy has consistently shown reduced FA in the uncinate fasciculus, bilaterally (Hoppenbrouwers et al. 2013; Lindner et al. 2017), and in the right hemisphere only (Craig et al. 2009; Motzkin et al. 2011; Sobhani et al. 2015; Sundram et al. 2012; Wolf et al. 2015) along with increased RD (Lindner et al. 2017) and MD (Sundram et al. 2012) in the uncinate fasciculus in individuals with psychopathy. The uncinate fasciculus is a long‐range fibre bundle that connects the orbitofrontal cortex to the anterior temporal lobes (including the amygdala and hippocampus) and, as such, is often associated with the limbic system (Von Der Heide et al. 2013). By virtue of this association, it has been suggested that abnormalities in the uncinate fasciculus (resulting in disrupted connectivity between the amygdala‐orbitofrontal cortex) may underpin some of the learning and memory deficits observed in individuals with psychopathy which, arguably, contribute to the characteristic cognitive and behavioural abnormalities in the disorder (Lindner et al. 2017; Von Der Heide et al. 2013). Previous DTI studies have also reported decreased FA in the corpus callosum (Sundram et al. 2012), anterior thalamic radiations (Hoppenbrouwers et al. 2013) and in the ventral and dorsal cingulum (Sethi et al. 2015), as well as increased radial diffusivity in the corpus callosum (Tesli et al. 2021) in individuals with psychopathy.

While previous studies have identified that abnormalities in white matter tracts could be related to psychopathy, results to date have been inconsistent. Craig et al. (2009) found a negative correlation between FA values in the uncinate fasciculus and PCL‐R factor 2 antisocial and lifestyle traits and the antisocial facet of the PCL‐R. More recent studies have reported findings in the same direction, but the associations relate to interpersonal and affective (Factor 1) traits. For example, both Lindner et al. (2017) and Wolf et al. (2015) report that reduced FA in the uncinate fasciculus was associated with the interpersonal/affective Factor 1 and interpersonal manipulation (IM) facet of psychopathy, not the antisocial factors. Vermeij et al. (2018) report negative correlations between Factor 1 traits and FA in white matter tracts connecting the temporal cortex, orbital cortex and amygdala. Negative correlations found between the affective facet and FA were specific to clusters in the right temporal lobe. While Hoppenbrouwers et al. (2013) found that FA values negatively correlated with scores on the PCL‐R interpersonal/affective Factor in the left orbitofrontal cortex and frontal pole, whereas deficits in the striato‐thalamo‐frontal network only correlated positively with the antisocial component. Overall, these studies indicate a need for further investigation into the distinct white matter microstructure correlates of psychopathy and its subcomponent facets.

Despite the increased use of DTI in the field, an overreliance on participants from institutional and clinical settings means there is less published research exploring whether these white matter associations hold across the full spectrum of psychopathy severity, for example, in the community, or if they are restricted to the more extreme presentation of the disorder. The few studies that have used community samples have observed similar results to those that use forensic or clinical samples. For example, Yang et al. (2005) investigated prefrontal grey and white matter volume in males from the community. They found positive associations between prefrontal and whole‐brain white matter volume and scores on Cooke and Michie's (2001) arrogant/deceptive factor along with increased prefrontal white matter and grey matter volume (GMV) in deceitful individuals (i.e., pathological liars) in comparison to both antisocial and normal control groups. Furthermore, the findings of reduced FA in the uncinate fasciculus from Sobhani et al. (2015) and Lindner et al. (2017), who both use community samples, are also in line with those of forensic populations.

Studying traits of personality disorders such as psychopathy in community samples provides a valuable perspective that is often underrepresented in clinical and forensic research (Skeem et al. 2011). Community sample approaches support dimensional models of psychopathy, allow for the identification of subclinical traits, including those that may have adaptive or maladaptive implications, and contribute to efforts aimed at characterising early risk markers (e.g., Viding and McCrory 2018). Moreover, investigating community‐based studies can reduce sampling bias, capture greater cultural and gender diversity and offer insights into how these traits impact everyday functioning outside of institutional settings. As such, they play an important role in advancing developmental models of psychopathy and informing public health and prevention strategies.

A frequent criticism of behavioural science is its reliance on samples from Western, Educated, Industrialised, Rich and Democratic (WEIRD) nations (Henrich et al. 2010; Rad et al. 2018). Indeed, most of what we know about psychopathy stems from studies conducted in Western nations, with a notable lack of systematic research focusing on regions outside the United States and Europe. This Western bias may hinder accurate understanding of personality disorders across different cultures, as disorders conceptualised and operationalised through Western individualistic psychological frameworks may not fully capture the complexities of personality disorders in other sociocultural contexts (Shou et al. 2021). This is of particular importance when considering psychopathic traits and the behaviours by which they manifest. Core characteristics of personality disorders are typically those that deviate from societal norms, thinking patterns and behavioural practices (Shou et al. 2021). However, research frequently overlooks the influence of sociocultural milieus and personal experiences in shaping these patterns and how cultural features may shape an individual's concept of self and others (Heine and Buchtel 2009; Triandis and Suh 2002). Moreover, evidence suggests that cognitive functions, lateralisation of processes, overall brain size and shape, neural activation patterns, hemispheric shape and the volume of neural structures differ as a function of culture (Hedden et al. 2008; Huang et al. 2019; Kitayama and Uskul 2011; Rozin et al. 2016; Yu et al. 2019; Zilles et al. 2013). These findings highlight the importance of investigating psychopathy's neural correlates in non‐Western populations.

A further issue concerns the lack of DTI research in women compared to male samples. To address this gap in the literature, Lindner et al. (2017) investigated white matter correlates of psychopathic traits in a sample of females from the community and found similar associations as those previously identified in male samples. Specifically, they found higher IM scores were negatively associated with uncinate fasciculus (FA and RD) and fornix (ad) integrity, while callous affect (CA) and ELs scores were negatively associated with white matter integrity in a cluster adjacent to the fusiform gyrus (FA). As is the case in previous research using male samples, the sample used in the Lindner et al. (2017) work was a single‐sex rather than a mixed‐sex sample. As such, the moderating effect of sex has yet to be tested in an adult sample. However, recent studies exploring white matter tracts in children with conduct disorder (CD) have observed sex differences in white matter alterations in the left corticospinal tract, fornix and internal capsule (Rogers et al. 2019) and right retrosplenial cingulum tract (González‐Madruga et al. 2020). Villemonteix et al. (2021) also demonstrated sex‐specific associations in children with CD between callous‐unemotional traits and ad in the uncinate fasciculus. Taken together, these findings highlight the importance of considering sex when investigating white matter microstructure in psychopathy.

Here, we used four commonly used metrics of white matter (FA, ad, RD and MD). As each metric reflects different aspects of white matter microstructure, using these metrics in combination may go some way to mitigate the potential pitfalls (e.g., crossing fibres) (Figley et al. 2022) associated with drawing inference from analyses using more global measures (e.g., FA and MD) and may provide a more a fine‐grained understanding of the white matter microstructure correlates of psychopathic traits. The primary aim of this work was to examine the main effects of psychopathic traits and their subcomponent facets on diffusion‐based measures of white matter microstructure (FA, RD, ad and MD) across the whole brain and in key regions of interest (ROIs)/tracts in Japanese males and females from the community. Based on recent evidence, we predicted that the severity of psychopathic traits and its subcomponent facets would be negatively associated with white matter integrity (reduced FA or ad and increased RD and MD) within the uncinate fasciculus (Hoppenbrouwers et al. 2013; Lindner et al. 2017), corpus callosum (Sundram et al. 2012; Tesli et al. 2021), anterior thalamic radiation (Hoppenbrouwers et al. 2013) and cingulum (Sethi et al. 2015). Furthermore, we aimed to test if sex influenced the strength and/or direction of such associations. While there are no white matter studies on psychopathy using adult mixed‐sex samples, data from several studies in adolescents with CD suggest white matter diffusivity may differ between males and females (Rogers et al. 2019; González‐Madruga et al. 2020; Villemonteix et al. 2021). In this context, while we expected some sex‐by‐psychopathy trait interactions in association with white matter microstructure, we made no a priori hypothesis in terms of the location of these associations. Given recent research showing that psychopathy factors or facets may be differentially and interactively associated with GMV (Chester et al. 2023; Miglin et al. 2022), the final aim was to investigate the interactive effects of psychopathy traits (factor and facet scores) on white matter structures.

## 2 Materials and Methods

### 2.1 Participants

Participants were 97 right‐handed adults aged 21–39 years (M = 27, SD = 5.3) (52 females) recruited from a Japanese work placement agency who took part in standardised psychometric testing and neuroimaging at Kyoto University, Japan. All participants were included in a previous structural imaging study exploring the grey matter structural neuroanatomical correlates of psychopathy traits (Chester et al. 2023). Full informed consent was obtained from participants, and ethical approval was obtained from the Ethics Committee at Kyoto University.

### 2.2 Psychopathic Traits Assessment

Psychopathic traits were measured using the *Self‐Report Psychopathy Scale Fourth Edition* (SRP‐4) Short Form (Paulhus et al. 2009). The SRP‐4 provides a total score for overall psychopathy and four subscale scores for each of the facets (ASB, CA, IM and EL). Responses on the CA and IM subscales were summed to create a ‘Factor 1’ score (14 items, α = 0.85) and responses on the ASB and EL subscales were summed to create a ‘Factor 2’ score (15 items, α = 0.76). Further details, including how the SRP‐SF has been validated in a Japanese community population, are outlined in previous work (Chester et al. 2023).

### 2.3 Diffusion‐Weighted Images Acquisition

DTI data were collected on a Siemens 3 T Verio scanner with a 32‐channel head coil at Kyoto University. Details of image acquisition are in Supporting Information.

### 2.4 DTI Data Preprocessing

Raw scans were visually examined by the author and a research assistant for image corruption, noise artefacts and excessive head movement before inclusion in the imaging pipeline. No scans were removed following quality assessment. Images were preprocessed using the FSL (FMRIB Software Library; V6) diffusion toolkit (Jenkinson et al. 2012). This included correction for motion and eddy‐current distortion and the creation of a binary brain mask using the brain extraction tool (BET). After which scalar maps of FA, ad, RD and MD were generated by fitting a diffusion tensor model at each voxel using the *DTIFit* tool in FSL. As previously outlined, as each metric reflects different aspects of white matter microstructure, using these measures in combination may help mitigate the potential limitation of drawing inferences from analyses that rely on these metrics in isolation (Figley et al. 2022).

### 2.5 Statistical Analysis

#### 2.5.1 Demographic Data and Sensitivity Analysis

Demographic analyses were performed in IBM SPSS Statistics version 27 (IBM Corp 2020). Independent samples *t*‐tests were conducted to compare SRP‐SF scores, age and total intracranial volume (TIV) between males and females. Levene's test indicated homogeneity of variances (all *p*'s > 0.05); thus, equal variances were assumed for all comparisons. Cohen's *d* was calculated to quantify the standardised effect size of mean group differences between males and females in SRP‐SF scores. A sensitivity analysis was conducted using the pwr.f2.test function from the *pwr* package in R (Champely 2020) to determine the minimum detectable effect size for a multiple linear regression model with four predictors. The analysis indicated that the study was powered to detect an effect size of *f* ^*2*^ = 0.13 (equivalent to *R* ^2^ ≈ 0.115) with 80% power at *α* = 0.05.

#### 2.5.2 Neuroimaging Data

##### 2.5.2.1 Whole Brain

Tract‐based Spatial Statistics (TBSS) were used to perform whole‐brain voxelwise statistical analyses of the data in FSL (Smith et al. 2007). Multiple linear regression analyses were performed to explore the associations between psychopathic traits and four commonly used quantitative biomarkers of white matter: FA, ad, RD and MD. The analyses tested for both positive and negative associations across total score, Factors 1 and 2 and each of the subfacet scores separately using permutation‐based analyses (5000 permutations). Threshold‐free cluster enhancement (TFCE) with *p* < 0.05 familywise error (FWE) correction was applied to account for multiple comparisons. Standardised regression coefficients (β*), adjusted *R*‐squared values (Adj. *R* ^2^) and 95% confidence intervals are reported to characterise the strength, direction and precision of associations (Egbuchulem 2022). Standardised coefficients were computed using the *lm.beta* package in R (Behrendt 2023). Anatomical locations for each significant cluster were labelled using the JHU DTI‐based white matter atlases (JHU‐ICBM‐DTI‐81 white matter labels atlas and JHU white matter tractography atlas; Hua et al. 2008; Mori et al. 2005; Wakana et al. 2007).

##### 2.5.2.2 Region of Interest

A priori ROIs were identified from previous literature identified by De Brito et al. (2021) regarding regions/tracts associated with psychopathy traits. These included the uncinate fasciculus, corpus callosum, anterior thalamic radiation and cingulum. Bilateral anatomical masks were created using the JHU‐ICBM‐DTI‐81 white matter atlas (Hua et al. 2008; Mori et al. 2005; Wakana et al. 2007). As above, the ROI analyses tested for positive and negative associations across the dimensional measures using permutation‐based analyses with TFCE thresholded at *p* < 0.05 FWE‐corrected. The *fslmeants* tool was used to extract whole brain and ROI level data and plot them to visualise any main or interacting effects.

For all main effect models, age and sex were included as covariates of no interest. A Factor 1 by Factor 2 interaction term was entered as a separate model. Furthermore, an interaction term between sex and psychopathy traits was included in the multiple linear regression analyses by setting up full factorial models. For all interaction models, age was included as a covariate of no interest.

## 3 Results

### 3.1 Demographic and Psychopathy Scores

Participant demographic information and SRP‐SF scores are reported as means and standard deviations (Table 1). The mean score for total psychopathic traits was 52 (SD = 13.7). There was no significant difference in total psychopathic trait score and subcomponent scores between males and females (all *p'*s > 0.05) except for EL, where males scored significantly higher than females (*p* = 0.012, *d* = 0.52). It is worth noting that the SRP‐SF scores reported here are generally comparable with those previously reported in community samples (Seara‐Cardoso et al. 2012) but below those from clinical and forensic samples (León‐Mayer et al. 2015; Neumann et al. 2015; Debowska et al. 2018).

**TABLE 1:** Sample characteristics: age, TIV and psychopathy scores.

	Total sample (*n* = 97)	Male (*n* = 45)	Female (*n* = 52)	
	M (±SD)	M (±SD)	M (±SD)	***p‐*value**
Age	27 (± 5.3)	27 (± 5.7)	27 (± 5.1)	0.933
TIV	1497 (±14.8)	1601 (±114.7)	1408 (±104.5)	**< 0.001**
**SRP‐4**				
Total psychopathy	52 (±13.7)	54 (±14.4)	50 (±12.9)	0.173
Interpersonal manipulation	14 (±5.2)	15 (±5)	14 (±5.4)	0.524
Callous affect	14 (±4.2)	14 (±4.4)	13 (±4.1)	0.327
Erratic lifestyle	14 (±4.1)	15 (±4.3)	13 (±3.8)	0.**012**
Antisocial behaviour	10 (±2.8)	10 (±3.4)	10 (±2.2)	0.731
Factor 1	28 (±8.6)	29 (±8.6)	27 (±8.7)	0.388
Factor 2	24 (±6)	25 (±6.6)	23 (±5.3)	0.060

*Note:* *p*‐values derived from *t*‐tests. There was no significant difference in total psychopathy and subcomponent scores between males and females (all *P*s > 0.05) except for erratic lifestyle, where males scored higher than females (*p* = 0.012, *d* = 0.52). It is worth noting that the SRP‐SF scores reported here are generally comparable with those previously reported in community samples (Seara‐Cardoso et al. 2012) but below those from clinical and forensic samples (León‐Mayer et al. 2015; Neumann et al. 2015; Debowska et al. 2018).

### 3.2 Neuroimaging Results

#### 3.2.1 Whole‐Brain Analysis

Across all participants, there were no significant positive or negative correlations between total psychopathic trait score, or its subcomponent facets, with any DTI measure (FA, ad, MD or RD). Furthermore, a whole‐brain factor‐by‐factor interaction analysis did not reveal any clusters of significant voxels.

**FIGURE 1:**
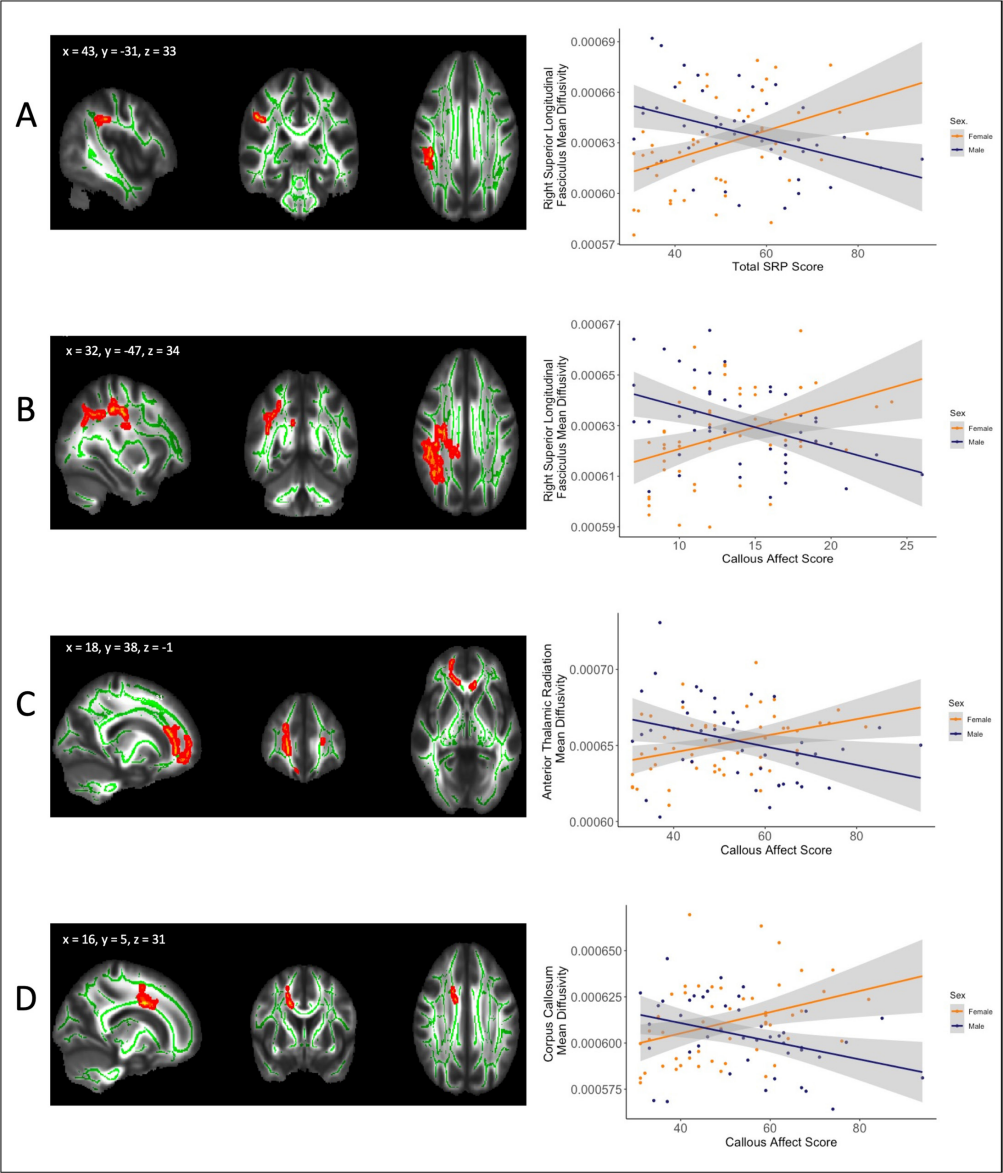
Sex interactions in white matter microstructure. (A) voxels within the right superior longitudinal fasciculus (x, y, z = 43, −31, 33) in which total psychopathy scores interacted with sex to predict mean diffusivity and voxels within the right (B) superior longitudinal fasciculus (x, y, z = 32, −47, 34), (C) anterior thalamic radiation (x, y, z = 18, 38, −1), (D) corpus callosum (x, y, z = 16, 5, 31) in which callous affect scores interacted with sex to predict mean diffusivity. For viewing purposes, skeletonised clusters have been ‘thickened’ using TBSSfill. Findings are overlaid onto the mean FA skeleton (green) in MNI space.

Total psychopathy scores interacted with sex to predict MD in the right superior longitudinal fasciculus (standardised *β* = −1.70, adj. *R* ^2^ = 0.16, 95% CI [−2.21 × 10^−6^, −8.10 × 10^−7^], *p* = 0.049) (Table 2, Figure 1A), such that total psychopathy scores were negatively correlated with MD in males, but positively correlated with MD in females.

**TABLE 2:** MNI atlas coordinates of clusters that showed a significant sex by psychopathy trait interaction in mean diffusivity.

		MNI coordinates (mm)						
Cluster label	Cluster size (voxels)	x	y	z	Corrected *p*	ICBM‐DTI‐81 white matter atlas label	JHU white matter tractography atlas/tracts within cluster	Region
**Total psychopathy (MD)**								
1	103	41	−28	33	0.049	Unclassified	Superior longitudinal fasciculus	Parietal lobe RH
**Callous affect (MD)**								
1	2044	44	−38	30	0.044	Unclassified	Superior longitudinal fasciculus/corticospinal tract/cingulum/anterior thalamic radiation	Frontal/parietal/occipital/limbic RH
2	1889	19	38	6	0.042	Anterior corona radiata	Anterior corona radiata/forceps minor/uncinate fasciculus/anterior thalamic radiation/inferior fronto‐occipital fasciculus/genu of corpus callosum	Frontal lobe LH/RH
3	489	31	42	−2	0.046	Unclassified	Inferior fronto‐occipital fasciculus/uncinate fasciculus/anterior thalamic radiation	Frontal lobe RH
4	207	14	3	31	0.048	Body of corpus callosum	Superior corona radiata/corpus callosum	Frontal lobe RH
5	73	19	23	25	0.049	Genu of corpus callosum	Anterior corona radiata/forceps minor/cingulum/body of corpus callosum	Frontal lobe/limbic RH
6	62	17	34	−17	0.049	Unclassified	Uncinate fasciculus/inferior fronto‐occipital fasciculus/anterior corona radiata	Frontal lobe RH

*Note:* Atlas coordinates of the maximum intensity voxel are reported.

Abbreviations: MD, mean diffusivity; MNI, Montreal Neurological Institute; RH/LH, right/left hemisphere; SRP‐SF, Self‐Report Psychopathy Scale Fourth Edition Short Form.

Furthermore, CA scores interacted with sex to predict MD in several clusters, including clusters in the right superior longitudinal fasciculus, anterior thalamic radiation, and within the body of the corpus callosum (*all p's* < 0.05) (Table 2, Figure 1B,C,D), such that CA scores were negatively correlated with MD in males but positively correlated with MD in females.

#### 3.2.2 ROI Analysis

**FIGURE 2:**
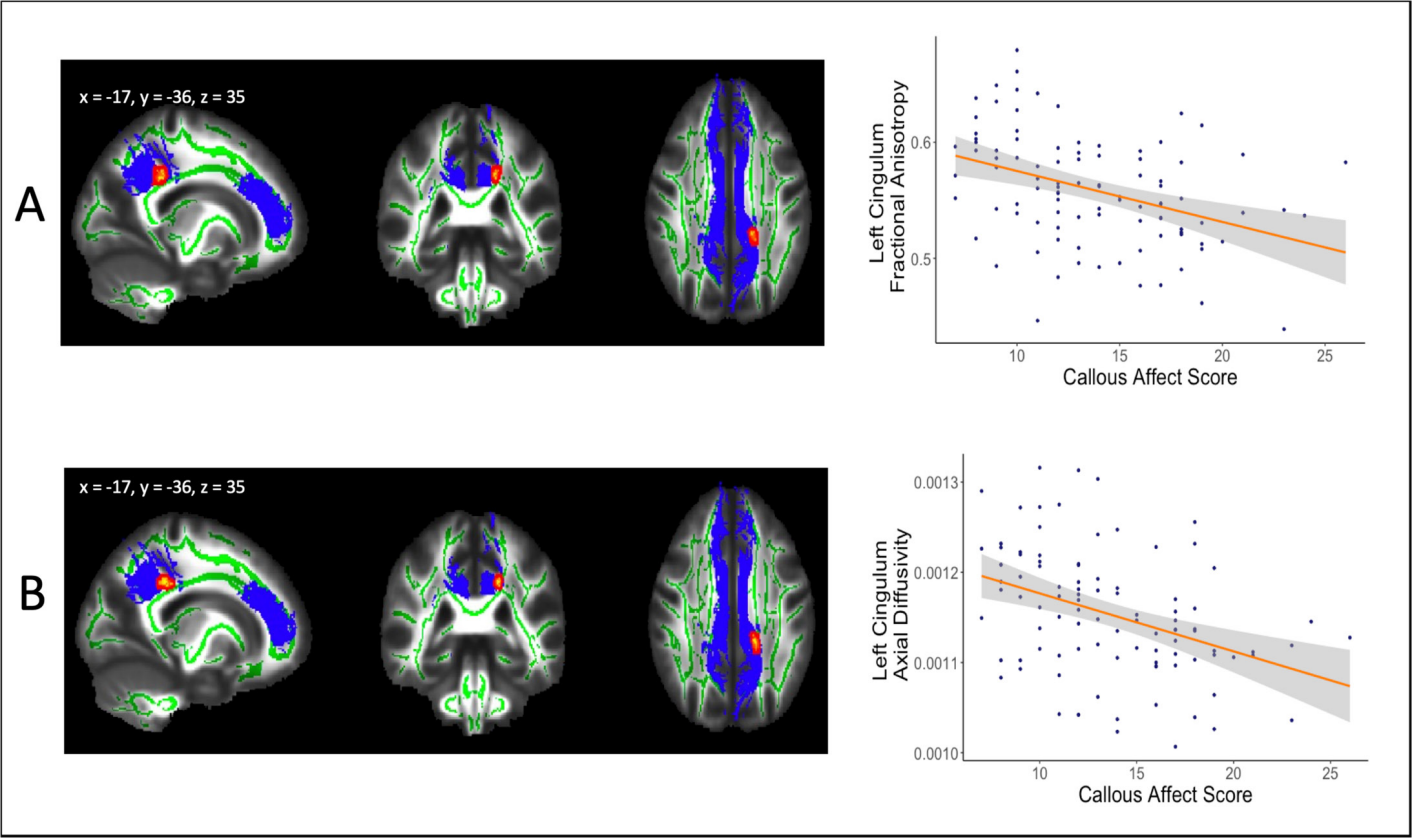
White matter microstructure in left cingulum. (A) voxels within the left cingulum (x, y, z = −17, −36, 35) in which callous affect scores were significantly negatively correlated with fractional anisotropy. (B) Voxels within the left cingulum (x, y, z = −17, −36, 35) in which callous affect scores significantly negatively correlated with axial diffusivity. For viewing purposes, skeletonised clusters have been ‘thickened’ using TBSSfill. Findings are overlaid onto the mean FA skeleton (green) in MNI space and cingulum mask (blue).

In the ROI analysis, we found significant correlations between psychopathy facet scores and white matter FA and ad in two clusters. Specifically, CA scores negatively correlated with FA (β* = −0.39, adj. *R* ^2^ = 0.14, 95% [CI −6.52 × 10^−3^, −2.26 × 10^−3^], *p* = 0.032) in one cluster in the left cingulum (Table 3, Figure 2A). Additionally, CA scores negatively correlated with ad in one cluster in the left cingulum (β* = −0.39, adj. *R* ^2^ = 0.14, 95% CI [−3.34 × 10^−6^, 9.52 × 10^−6^], *p* = 0.027) (Table 3, Figure 2B). No further main or interaction effects were observed within any other ROIs for any DTI index.

**TABLE 3:** MNI atlas coordinates of clusters significantly associated with SRP‐4 callous affect scores.

		MNI coordinates (mm)						
Cluster label	Cluster size (voxels)	x	y	z	Corrected *p*	ICBM‐DTI‐81 white matter atlas label	JHU white matter tractography atlas/tracts within cluster	Region
**Fractional anisotropy**								
1	41	−18	−41	32	0.032	Unclassified	Cingulum/posterior corona radiata	Limbic LH
**Axial diffusivity**								
1	43	−17	−37	36	0.027	Posterior corona radiata	Cingulum/posterior corona radiata/anterior thalamic radiation	Parietal/limbic LH

*Note:* Atlas coordinates of the maximum intensity voxel are reported.

Abbreviations: MD, mean diffusivity; MNI, Montreal Neurological Institute; RH/LH, right/left hemisphere; SRP‐SF, Self‐Report Psychopathy Scale Fourth Edition Short Form.

## 4 Discussion

In this study, we used DTI to investigate associations between white matter microstructure and self‐reported psychopathic traits at a whole‐brain level and within specific ROIs. In addition, we aimed to establish if sex influenced the strength or direction of such associations. The final aim was to investigate the interactive effects of psychopathy traits (factor/facet scores) on white matter.

In partial support of our hypothesis, the ROI analyses demonstrated that higher CA scores were associated with reduced FA and ad in the left cingulum. In addition, our whole‐brain analysis revealed that sex moderates the association between psychopathy severity (total psychopathy and CA scores) and white matter integrity, such that, in the superior longitudinal fasciculus, anterior thalamic radiation and corpus callosum, total psychopathy and CA scores were negatively associated with MD in males, whereas the association was positive in females.

In partial support of our hypothesis, the ROI analysis showed that CA scores were inversely correlated with FA in the left cingulum. This effect may be primarily driven by abnormalities in axonal structural integrity within this region, as we also found CA scores were negatively correlated with ad in the left cingulum. The cingulum is a complex association fibre bundle that connects frontal, parietal and medial temporal regions and is centrally positioned within the default mode network (Van Den Heuvel et al. 2008). As such, it is possible that reduced axonal integrity in the cingulum could impair functional connectivity between these regions, which are implicated in empathy, moral reasoning and self‐referential processing (Andrews‐Hanna et al. 2014; Smallwood et al. 2021; Van Den Heuvel et al. 2008). Disruptions in these connections may hinder an individual's ability to evaluate the emotional states of others and reflect on the moral consequences of their actions, both of which are processes frequently disrupted in individuals with elevated callous‐unemotional traits (Marsh et al. 2013; Viding and McCrory 2019). Furthermore, given that the cingulum continues to develop into early adulthood (Lebel et al. 2008), reduced axonal integrity may reflect atypical neurodevelopmental trajectories that potentially limit the maturation of social cognition systems. Such disruptions may impair the processing of moral and social feedback, reducing sensitivity to punishment or disapproval, which in turn may contribute to the behavioural characteristics frequently observed in individuals with elevated CA scores (Blair 2007; Frick and White 2008). It is noteworthy that we found reduced white matter integrity was specifically related to the CA facet (Factor 1) of psychopathy. Arguably, it is this core emotional impairment of the psychopathic personality (lack of empathy, glibness, manipulativeness, etc.) that distinguishes it from other antisocial disorders such as antisocial personality disorder (ASPD).

Although we note that these results differ from some published studies using male prisoner (Wolf et al. 2015) and female community (Lindner et al. 2017) samples, this finding is, however, in line with the work of Sethi et al. (2015), who observed significantly lower FA in the left dorsal cingulum and bilateral ventral cingulum (but no difference in RD) in individuals with psychopathy compared to healthy controls. Interestingly, within the group of prisoners with psychopathy, PCL‐R Factor 1 scores negatively correlated with FA in the cingulum bilaterally. As such, they propose a ‘dual network’ model of psychopathy, which posits that the main diagnostic features of psychopathy, namely, the affective‐interpersonal (Factor 1) traits and lifestyle‐antisocial (Factor 2) behaviours, are dissociable at a network level. Indeed, our findings suggest that there may be distinct neuroanatomical characteristics of Factor 1 traits of psychopathy. Our study adds to accumulating research demonstrating alterations within the default mode network in psychopathy, particularly Factor 1 scores (Anderson et al. 2018; Deming and Koenigs 2020; Espinoza et al. 2018; Johanson et al. 2020). Here, we add to this body of research by pointing away from myelination degradation as an explanation and instead implicating reduced axonal structural integrity as underpinning the deficits evidenced in previous research. We should note, however, that while several studies have reported associations between psychopathy and disruptions in the DMN, a recent study in a community sample found no significant relationship between psychopathic traits and DMN activity (Bakiaj et al. 2025). It must be noted, however, that the psychopathy measure in that study only had 9 items and had very poor internal consistency (0.59).

To our knowledge, this study is the first to show that sex moderates the association between psychopathic traits and white matter microstructural integrity in several major white matter tracts, namely, the superior longitudinal fasciculus, anterior thalamic radiation and corpus callosum. In detail, we found total psychopathy scores interacted with sex to predict MD in the right superior longitudinal fasciculus. Furthermore, we show that CA scores interacted with sex to predict MD in several clusters, including clusters in the right superior longitudinal fasciculus, anterior thalamic radiation and within the body of the corpus callosum. Across all significant clusters discussed below, psychopathy trait scores were positively correlated with MD in females, whereas the opposite pattern was observed in males.

While atypical white matter microstructure in the superior longitudinal fasciculus has not been previously linked to adult psychopathy, there have been reports of reduced superior longitudinal fasciculus white matter integrity (FA, RD and MD) in adult patients with ASPD (Jiang et al. 2017), individuals with intermittent explosive disorder (Lee et al. 2016), violent individuals and those with psychosis (Tesli et al. 2021). Research has also shown that reduced white matter integrity in the superior longitudinal fasciculus is associated with risky behaviour (Jiang et al. 2017) and aggressive acts (Karlsgodt et al. 2015). This association is also consistent with previous findings in youths where atypical white matter structure (FA, RD and MD) in the superior longitudinal fasciculus has been reported in children with CD (Decety et al. 2015; Haney‐Caron et al. 2014; Li et al. 2005), including those with high levels of callous‐unemotional traits (Puzzo et al. 2018).

The superior longitudinal fasciculus is an extensive association tract, made up of three branches, connecting the frontal lobe and widespread areas of the parietal, occipital and temporal lobes (Janelle et al. 2022). This tract is important for several cognitive and emotion regulatory functions that are impaired in psychopathy, namely, social information processing, working memory, emotion processing and control/inhibition of emotions and impulsive actions (de Schotten et al. 2011; Klarborg et al. 2013; Nakajima et al. 2020; Parlatini et al. 2017). As such, our results could indicate that abnormalities in superior longitudinal fasciculus connectivity may give rise to difficulties in emotion regulation and self‐control and thus contribute to the risk‐taking and impulsive behaviours commonly associated with psychopathy (De Brito et al. 2021).

Sex and psychopathy also interacted to predict MD in the corpus callosum, which connects brain regions across the two cerebral hemispheres. This finding is in line with existing DTI literature reporting reduced white matter integrity in the corpus callosum in association with psychopathy. For example, Sundram et al. (2012) report reduced FA and increased MD in association with psychopathy scores, while Tesli et al. (2021) report a positive association between RD and PCL‐R scores in violent offenders. Notably, Lindner et al. (2017) observed reduced ad in the corpus callosum in females with CD compared with a female healthy control group. It is also in accord with studies reporting structural and functional abnormalities in the corpus callosum in association with psychopathy (Hoppenbrouwers et al. 2013; Raine et al. 2003), as well as research on white matter microstructure in youths, which found higher ad, reduced RD, and MD in those with CD compared to typically developing youths (Rogers et al. 2019).

The corpus callosum is the largest white matter structure in the brain with both intra‐ and interhemispheric projections and is fundamental to the exchange of information and the effective coordination of processes across the hemispheres, performing both excitatory and inhibitory functions on the contralateral hemisphere (Bloom and Hynd 2005; van der Knaap and Ham 2011). It is also important for regulating emotion, attention and arousal (Raine et al. 2003; Sundram et al. 2012). Lesions of the corpus callosum have been postulated to contribute to impairments in attention and processing speed along with diminished social and emotional processing (Anderson et al. 2017; Huynh‐Le et al. 2021; Gazzaniga 2000).

Atypical white matter microstructural integrity in the corpus callosum is believed to cause deficits in interhemispheric communication and thus leads to an imbalance/asymmetry between the hemispheres (Kelley et al. 2017). This notion is supported by findings of increased interhemispheric transfer time (i.e., the time it takes for information to be communicated through the corpus callosum) in individuals with psychopathy (Hiatt and Newman 2007). While speculative, it could be that inefficient/reduced inhibition of contralateral hemispheric activity could contribute to the promotion of behaviours normally modulated by the opposite hemisphere. For example, neuroimaging and behavioural studies report that the left frontal cortex is associated with processes related to approach motivation, while the right is associated with avoidance motivation. A disruption in interhemispheric inhibition, particularly where the right hemisphere is unable to inhibit the left, may result in increased impulsivity, a tendency toward immediate gratification and approach‐related anger (Schutter and Harmon‐Jones 2013). The present findings provide some support for the premise that dysfunctional connectivity in the corpus callosum, and the resulting hemispheric asymmetries, may be related to psychopathy.

The anterior thalamic radiation result aligns with previous DTI studies that report reduced white matter integrity (lower RD, MD and increased FA) in individuals with psychopathy (Hoppenbrouwers et al. 2013; Tesli et al. 2021), ASPD (Sundram et al. 2012) and in youths with CD (Haney‐Caron et al. 2014; Puzzo et al. 2018; Rogers et al. 2019) within this tract. While these earlier studies report white matter anterior thalamic radiation differences bilaterally, the findings of the current study are specific to the right hemisphere. The anterior thalamic radiation, which forms part of the limbic system, is one of four distinct pathways of thalamocortical radiations (fibres) that connect the thalamus to the cerebral cortex (Catani et al. 2013; Kouakou et al. 2017). It is composed of reciprocal fibres that run through the anterior limb of the internal capsule and connect the anterior and mediodorsal thalamic nuclei to the dorsolateral and ventrolateral prefrontal cortices, orbitofrontal and anterior cingulate cortices (Jang and Yeo 2014). The anterior thalamic radiation has been associated with cognitive functions that are impaired in individuals with psychopathy (Hare and Neumann 2009; Gillespie et al. 2022), namely, inhibitory control (Koini et al. 2017), empathy (Parkinson and Wheatley 2014) and emotion recognition (Koevoets et al. 2022). This result highlights the importance of anatomical connections between sensory and limbic regions that may be particularly relevant to the psychopathic personality.

A notable difference between our findings and previous work is the sex differences observed in the present study. Our results provide support for the hypothesis that psychopathic traits may develop through distinct neurodevelopmental trajectories in males and females. Recent neuroscience work suggests that in women, comparable traits may emerge from more distributed or functionally distinct circuitry, shaped by both neurobiological and sociocultural influences (Tully et al. 2021). Consistent with this, data from several studies suggest that white matter development differs by sex in terms of timing, regional patterning and hormonal sensitivity, which may give rise to divergent neural substrates for similar behavioural traits in males and females (Herting et al. 2012; Kurth et al. 2021; Simmonds et al. 2014; Wang et al. 2012). Females generally show earlier onset of white matter microstructural maturation, including myelination and pruning, whereas males tend to exhibit more protracted white matter development extending into early adulthood (Buyanova and Arsalidou 2021; Kaczkurkin et al. 2019). Given this, increased MD in females may reflect atypical white matter organisation, such as reduced axonal density or coherence, which could impair typical emotional and social functioning. In contrast, lower MD in males may reflect more tightly packed or selectively pruned white matter pathways, potentially supporting the characteristic emotional detachment and diminished affective responsiveness observed in individuals with elevated psychopathic traits. These sex‐specific differences may result from differential timing of neurodevelopmental processes, such as more extensive and delayed myelination or pruning processes in males (Kaczkurkin et al. 2019; Schmithorst et al. 2008). In addition, socialisation processes may lead to less overt or more internalised expressions of psychopathy in women and the engagement of alternative regulatory systems (Tully et al. 2021; Cale and Lilienfeld 2002). It is possible, therefore, that if psychopathic traits are expressed through more distributed or compensatory circuits in females, this may contribute to less efficient or more disorganised white matter microstructure (characteristics often reflected in increased MD). As such, elevated MD may represent a neurodevelopmental signature of alternative trait regulation pathways in females, shaped by both biological and social influences. Further research could explore this possibility in greater depth, as our data cannot directly examine the developmental mechanisms involved.

We previously reported that psychopathy and its subcomponent traits were associated with grey matter and that sex acted as a moderator in those associations (Chester et al. 2023). The current study builds on and extends this work to suggest that, as well as sex‐specific grey matter correlates, white matter microstructure/connectivity correlates of psychopathy may also differ by sex. As such, it provides further support for sex‐specific brain correlates of psychopathy, which may help us to understand the sexually dimorphic expressions of psychopathy outlined in the literature (e.g., Fontaine et al. 2010; Tully et al. 2021).

Previous adult DTI studies of psychopathy have used either male or female samples, and as such, this sex difference is novel. However, our work is broadly consistent with that of Decety et al. (2015) who used a mixed‐sex sample of children. They showed that CD symptoms were associated with greater RD in several areas, including the right superior longitudinal fasciculus and anterior thalamic radiation, and this association was stronger in females than in males. Although our work supports sex‐specific effects of psychopathy on white matter microstructure, these findings must be interpreted with caution given their novelty, and future research specifically designed to compare neuroanatomical correlates of the disorder between males and females needs to be undertaken to validate these findings.

Notably, care must be taken when interpreting diffusion parameters. There are well‐known difficulties in interpreting findings from DTI given the complex microstructural architecture that may be present in each image voxel. Such ‘crossing fibre’ populations include areas where fibres may be splaying, crossing, ‘kissing’, kinking and twisting (Vos et al. 2012). In addition, changes in diffusion anisotropy could be attributed to other components within white matter such as formations of glial cells and ‘ectopic’ neurons found in white matter (Concha 2014). This is particularly important when considering the MD results. The measure of MD indexes the averaged magnitude of diffusion, regardless of directionality (Concha 2014), and therefore provides a somewhat indirect measure of white matter integrity. It is unclear if changes in MD reflect abnormalities of axon structure (e.g., axonal injustice, incoherent orientation of axons and reduced axonal packing density) or myelin degradation (e.g., disruption of myelin sheath). It has also been reported that MD values decrease as the angle between fibre populations increases and are lower in regions where there are fibre populations with more than one dominant orientation (Vos et al. 2012).

A further potential limitation of the current study is that psychopathy was assessed using a shortened, self‐report measure. Such a measure is unlikely to approximate the in‐depth and exhaustive PCL‐R and is open to deception. It is important to bear this in mind when comparing self‐report results with those where trained clinicians have assessed psychopathy. Despite this, the use of a large mixed‐sex community sample represents a strength of our work, which allows for the study of neuroanatomical associations throughout the brain in healthy individuals with varying levels of psychopathic tendencies. In addition, while the SRP‐SF has been validated in a Japanese community sample, we acknowledge that diverse sociocultural factors may lead to variation in the expression and interpretation of psychopathic traits. Future research should prioritise cultural awareness and explore the cultural appropriateness and conceptual equivalence of psychopathy measures in non‐Western populations. Finally, the absence of additional socioeconomic status or education level data somewhat limits generalisability and constrains our ability to characterise fully participants' socio‐occupational functioning. Nonetheless, all participants self‐reported no history of neurological or psychiatric conditions, and SRP‐SF scores were broadly consistent with those documented in prior community‐based research.

In summary, we demonstrate that reduced FA and ad within the left cingulum are associated with total psychopathy score as well as the subcomponent facet of CA. The second major finding was that, in a sample of non‐Western healthy adults, sex moderates the association between psychopathic traits and white matter microstructural integrity in several major white matter tracts. Finally, a further key finding of this study was the discovery of associations between white matter integrity and psychopathy traits in a tract not previously indicated in adult DTI studies, namely, the superior longitudinal fasciculus. Taken together, those findings significantly extend our understanding of white matter microstructure in psychopathy.

## Author Contributions

**S. C. Chester:** conceptualization, formal analysis, visualization, writing – original draft. **J. C. Rogers:** conceptualization, formal analysis, supervision, writing – review and editing. **T. Ogawa:** investigation. **M. Terao:** investigation. **R. Nakai:** investigation. **N. Abe:** conceptualization, funding acquisition, investigation, resources, writing – review and editing. **Stephane De Brito:** conceptualization, funding acquisition, investigation, project administration, supervision, writing – review and editing.

## Ethics Statement

Ethical approval was obtained from the Ethics Committee at Kyoto University. Full informed consent was obtained from all individual participants included in the study.

## Conflicts of Interest

The authors declare no conflicts of interest.

## Peer Review

The peer review history for this article is available at https://www.webofscience.com/api/gateway/wos/peer‐review/10.1111/ejn.70177.

## Supporting information


**Data S1.** Supporting Information.

## Data Availability

The data underlying the article will be shared on reasonable request to the corresponding authors.
