# Supplementary material for: White Matter Correlates of Psychopathic Traits in a Japanese Community Sample: Sex Matters!
Source: Eur J Neurosci. 2025 Jul 14;62(1):e70177. doi: 10.1111/ejn.70177 (PMC12257594; doi:10.1111/ejn.70177)
Supplement: Supplementary file 1 — Data S1. Supporting Information. [file EJN-62-0-s001.pdf]

Supplementary Material

Diffusion-weighted images acquisition

For diffusion-weighted imaging, images were acquired with diffusion gradients (b-value = 1500 s/mm²) applied in 64 directions. In addition, two b-value = 0 (s/mm²) volumes with reversed phase encoding (blip-up/blip-down) were acquired yielding pairs of images with distortions in opposite phase-encode directions to enable estimation of susceptibility-induced distortions (repetition time (TR) = 8950ms, echo time (TE) = 87ms, slice thickness = 2.0 mm, flip angle = 90 degrees, field of view = 256mm, voxel size 2 x 2 x 2mm) yielding 72 axial slices). Duration of the scan was 10 minutes and 2 seconds. DTI data was acquired during a sequence comprised of structural and functional scans, some of which have been previously reported in other papers (Chester et al., 2023).
